# French *Aedes albopictus* are able to transmit yellow fever virus

**DOI:** 10.2807/1560-7917.ES.2016.21.39.30361

**Published:** 2016-09-29

**Authors:** Fadila Amraoui, Marie Vazeille, Anna Bella Failloux

**Affiliations:** 1 Institut Pasteur, Arboviruses and Insect Vectors, Department of Virology, Paris, France

**Keywords:** *Aedes albopictus*, Yellow fever, Vector competence

## Abstract

We assessed the ability of a French population of *Aedes albopictus* to transmit yellow fever virus (YFV). Batches of 30 to 40 female mosquitoes were analysed at 7, 14 and 21 days post-exposure (dpe). Bodies, heads and saliva were screened for YFV. Infectious viral particles were detected in bodies and heads at 7, 14 and 21 dpe whereas the virus was found in saliva only from 14 dpe. Our results showed that *Ae. albopictus* can potentially transmit YFV.

We assessed the vector competence of *Aedes albopictus* collected in France for a West African strain of yellow fever virus (YFV). Our results show that this temperate population of *Ae. albopictus* was able to deliver virus through saliva 14 days after receiving an infectious blood-meal.

## Experimental infection of mosquitoes

A YFV S79-P4 strain isolated in 1979 from a human case in Senegal [1] was passaged twice on newborn mice and two times on C6/36 *Ae. albopictus* cells. Viral stocks were produced on C6/36 *Ae. albopictus* cells.

*Ae. albopictus* mosquitoes used for the study originated from Bar-sur-Loup, a commune in the department of Alpes-Maritimes, which is in the region of Provence-Alpes-Côte d'Azur in south-east France. Eggs were collected from the field in ovitraps and reared in an insectary for 11 generations (the generation time is approximately 10 days) before experimental infections. Several batches of 200 larvae were reared in pans containing 1 L of dechlorinated tap water and a yeast tablet renewed every two days. Adults were maintained at 28 °C ± 1 °C in 80% relative humidity with a light:dark cycle of 16h:8h. The mosquitoes were fed ad libitum with a 10% sucrose solution. Females were blood fed three times a week on anaesthetised mice (OF1 mice, Charles River laboratories, France). Adult females were exposed to an infectious blood-meal containing 10^6.2^ foci fluorescent units (FFU)/mL of YFV S79-P4 strain mixed with rabbit blood and maintained at 28 °C for 21 days without any additional blood meals.

A total of 30 to 40 exposed mosquitoes were analysed at 7, 14 and 21 days post-exposure (dpe) to estimate the four indices describing the vector competence: (i) the infection rate (IR), which corresponds to the proportion of successfully infected mosquitoes (viral particles detected in bodies) after exposure to an infectious blood-meal among analysed mosquitoes, (ii) the disseminated infection rate (DIR), which measures the proportion of mosquitoes with evidence that the virus crossed the midgut barrier to reach the haemocoel and infected internal organs (infection detected in heads) among infected mosquitoes, (iii) the transmission rate (TR), which estimates the proportion of mosquitoes with the virus present in saliva among mosquitoes able to disseminate the virus in the mosquito haemocoel (examined when calculating DIR), and (iv) the transmission efficiency (TE), which corresponds to the overall proportion of females with the virus present in saliva among the total number of tested mosquitoes. Saliva was collected using the forced salivation technique previously described [2]. Briefly, wings and legs of each mosquito were removed from each mosquito and the proboscis was inserted into a 20 μL tip containing 5 μL of fetal bovine serum (FBS). After 30 to 45 min of salivation, FBS containing saliva was expelled in 45 μL of Dulbecco's modified Eagle medium (DMEM) for further titration. Heads/bodies homogenates and saliva from respective mosquitoes were titrated by focus fluorescent assay on C6/36 *Ae. albopictus* cells as prior described [3].

## Vector competence analysis

When analysing the ability of *Ae. albopictus* to be infected at 7, 14 and 21 dpe, IRs remained below 15/40 and were similar regardless of the dpe examined (7dpe: 6/40, 14 dpe: 15/40 and 21 dpe: 8/30; Fisher’s exact test: p = 0.074). When testing the ability of mosquitoes to undergo dissemination of the virus beyond the midgut barrier, DIR did not exceed 6/8 as observed at 21 dpe and remained comparable for the three dates post-exposure (7 dpe: 2/6, 14 dpe: 9/15 and 21 dpe: 6/8; Fisher’s exact test: p = 0.29).

When examining mosquito saliva for YFV among mosquitoes with a viral dissemination to calculate the TR, we found that the virus could be detected in saliva at 14 dpe (TR=2/9) and 21 dpe (TR=1/6). No virus was detected at 7 dpe. The corresponding TEs for *Ae. albopictus*, which take into account the total number of tested mosquitoes, were two individuals among 40 tested at 14 dpe and one among 30 at 21 dpe. When considering only mosquitoes with infectious saliva (n=3), a mean of 52 viral particles (standard deviation ± 28; n=2 individual mosquitoes’ saliva examined) was estimated at 14 dpe and 10 viral particles (1 mosquito’s saliva) at 21 dpe. Hence *Ae. albopictus* from southern France was able to transmit a West African YFV from 14 dpe. 

In a separate unpublished study (data not shown) that we conducted on *Ae. aegypti*, we found that at 14 dpe, *Ae. aegypti* had an IR of 5/17, a DIR of 2/5 and a TE of 2/17. This may suggest that *Ae. albopictus* mosquitoes might have higher rates of infection and dissemination of the virus in the body (15/40 and 9/15 respectively) than *Ae. aegypti*, albeit a lower TE (2/40).

## Background

Yellow fever (YF) is a potentially deadly disease with symptoms including jaundice, enlargement of the liver, and haemorrhage [4]. It is caused by YFV (*Flavivirus, Flaviviridae)*, a virus that was first isolated in West Africa in 1927 [5]. Globally, the heaviest burden of YF is in Africa where the endemic area covers 34 countries and concerns ca 500 million people [6].

Besides genetic differences between seven YFV genotypes identified to date [7], the competence of potential mosquito vectors to transmit the virus may affect the distribution pattern of YF outbreaks. In sub-Saharan Africa, where more than 90% of YF cases occur, three different transmission cycles have been described [4]. In the jungle cycle, YFV can spread between non-human primates by canopy-dwelling mosquitoes such as *Ae. africanus*. The intermediate or savannah cycle involves other mosquito species including *Ae. luteocephalus, Ae. furcifer, Ae. metallicus, Ae. opok, Ae. taylori, Ae. vittatu*s and members of the *simpsoni* complex. In areas where this cycle occurs, termed ‘zones of emergence’, YFV is transmitted from non-human primates to humans. Lastly, the urban cycle involves transmission of YFV between humans by the anthropophilic mosquito *Ae. aegypti*. In South America, YFV circulates exclusively in a jungle cycle involving *Haemagogus janthinomys* and *Sabethes chloropterus* mosquitoes and non-human primates [4]. The virus is absent in Asia although local *Ae. aegypti* are susceptible to the virus [8].

Since 1937, YF can be prevented through immunisation provided by the 17D vaccine; one dose confers a protective immunity for life and more than 650 million doses have been distributed in the past 75 years [9]. In endemic areas for YF however, funds are lacking to stimulate YFV vaccine production and accelerate vaccination campaigns, and human cases continue to be recorded annually. Moreover, during the past 20 years, at least one annual YF outbreak has been reported in Africa, mainly in West Africa (East and Central African countries are usually less affected). In such outbreaks, human cases are mainly associated with mass migrations of non-immunised people who have been exposed to YF in endemic areas, reminding that YF is still a major public health problem.

On 21 January 2016, an outbreak of YF occurred Angola [10]. With more than 3,000 suspected cases and 300 deaths as of 10 June 2016, the country is facing the most important urban YF outbreak observed so far in Africa [11]. Despite a slow decrease in the number of cases in Angola since the end of March 2016 [12], YFV circulation meanwhile continued to expand to neighbouring countries, such as Congo [13] and Uganda [14]. In Congo, 700 suspected cases with 63 deaths were recorded on 31 May 2016 while in Uganda, 30 cases including seven deaths were reported from 26 March to 18 April 2016. Most cases were found in cities suggesting that transmission implicates urban vectors, mainly *Ae. aegypti.* Imported YF cases from Angola were also later confirmed in Kenya [15] and China [16,17], highlighting that while the YF vaccine is very effective, there is a potential risk for unvaccinated travellers from endemic areas to further export the virus.

## Discussion

The establishment of a local YF transmission cycle outside endemic areas is related to competent *Aedes* mosquitoes, active all year long in tropical regions and during the warm period in temperate areas. The mosquito species *Ae. albopictus* is present in 20 European countries [18], and a strain of this species (Houston) in the United States has been previously reported to be a competent vector for YFV [19]. Hence travellers returning to Europe from countries where a YF outbreak is occurring could be a source of infection for local strains of *Ae. albopictus*. We therefore assessed the competence of *Ae. albopictus* mosquitoes from the south of France for a West African strain of YFV. 

The virus was detected at 14 dpe in saliva of the French *Ae. albopictus* mosquitoes at a rate of two mosquitoes in 40, a relatively low TE. While this is reassuring, a low vector competence can on the other hand contribute to select for virulent virus strains capable of eliciting high viraemia in humans [20] and causing more severe clinical symptoms [21]. Moreover although our results point to a low TR (2/9) for YFV, the anthropophilic nature of *Ae. albopictus* mosquitoes and their high densities in urban areas may allow them to be a vector of YFV. 

Concerning the virus strain assessed in this study, the West African YFV strain should not be very genetically distant from the other six genotypes with ca 9% amino-acid divergence between strains, indicating genetic stability of YFV genotypes [7]. However, small genetic changes in the viral genome may change the vector competence.

As Europe has faced YF outbreaks in the past [22], the last being recorded in Gibraltar in 1905, a risk of importation of YF into Europe is to be considered. Although so far there have been hardly any reports from Europe of imported YF cases, many imported cases of chikungunya and dengue, two other arboviral diseases, have been documented [23]. If YF follows the same path as dengue and chikungunya, which have a greater number of imported cases, a local transmission of YF in temperate regions where *Ae. albopictus* is established becomes a plausible scenario, underlining the need for continued vigilance for YF. 

## References

[1] Rodhain F Hannoun C Jousset FX Ravisse P Isolement du virus de la fièvre jaune à Paris A partir de deux cas humains importés [Isolation of the yellow fever virus in Paris from 2 imported human cases]. Bull Soc Pathol Exot Filiales. 1979;72(5-6):411-5. 261928

[2] Dubrulle M Mousson L Moutailler S Vazeille M Failloux AB Chikungunya virus and Aedes mosquitoes: saliva is infectious as soon as two days after oral infection. PLoS One. 2009;4(6):e5895. 10.1371/journal.pone.0005895 19521520 PMC2690823

[3] Vazeille M Yébakima A Lourenço-de-Oliveira R Andriamahefazafy B Correira A Rodrigues JM Oral receptivity of Aedes aegypti from Cape Verde for yellow fever, dengue, and chikungunya viruses. Vector Borne Zoonotic Dis. 2013;13(1):37-40. 10.1089/vbz.2012.0982 23199267

[4] Barrett AD Monath TP Epidemiology and ecology of yellow fever virus. Adv Virus Res. 2003;61:291-315. 10.1016/S0065-3527(03)61007-9 14714435

[5] Barrett AD Higgs S Yellow fever: a disease that has yet to be conquered. Annu Rev Entomol. 2007;52(1):209-29. 10.1146/annurev.ento.52.110405.091454 16913829

[6] Vasconcelos PF Monath TP Yellow Fever Remains a Potential Threat to Public Health. Vector Borne Zoonotic Dis. 2016;16(8):566-7. 10.1089/vbz.2016.2031 27400066

[7] Mutebi JP Wang H Li L Bryant JE Barrett AD Phylogenetic and evolutionary relationships among yellow fever virus isolates in Africa. J Virol. 2001;75(15):6999-7008. 10.1128/JVI.75.15.6999-7008.2001 11435580 PMC114428

[8] Tabachnick WJ Wallis GP Aitken TH Miller BR Amato GD Lorenz L Oral infection of Aedes aegypti with yellow fever virus: geographic variation and genetic considerations. Am J Trop Med Hyg. 1985;34(6):1219-24. 3834804 10.4269/ajtmh.1985.34.1219

[9] Barrett AD Yellow Fever in Angola and Beyond--The Problem of Vaccine Supply and Demand. N Engl J Med. 2016;375(4):301-3. 10.1056/NEJMp1606997 27276108

[10] Grobbelaar AA Weyer J Moolla N Jansen van Vuren P Moises F Paweska JT Resurgence of Yellow Fever in Angola, 2015-2016. Emerg Infect Dis. 2016;22(10):1854-5. 10.3201/eid2210.160818 27536787 PMC5038398

[11] World Health Organization (WHO). Yellow fever – Angola. Geneva: WHO; 14 Jun 2016. Available from: http://www.who.int/csr/don/14-june-2016-yellow-fever-angola/en/

[12] World Health Organization (WHO). Situation report. Yellow fever. 15 July 2016. Geneva: WHO; 15 Jul 2016. Available from: http://apps.who.int/iris/bitstream/10665/246224/1/yellowfeversitrep-15Jul16-eng.pdf?ua=1

[13] World Health Organization (WHO). Yellow fever – Democratic Republic of the Congo. Geneva: WHO; 2 Jun 2016. Available from: http://www.who.int/csr/don/02-june-2016-yellow-fever-drc/en/

[14] World Health Organization (WHO). Yellow fever – Uganda. Geneva: WHO; 2 May 2016. Available from: http://www.who.int/csr/don/02-may-2016-yellow-fever-uganda/en/

[15] Vasconcelos PF Monath TP Yellow Fever Remains a Potential Threat to Public Health. Vector Borne Zoonotic Dis. 2016;16(8):566-7. 10.1089/vbz.2016.2031 27400066

[16] Chen Z Liu L Lv Y Zhang W Li J Zhang Y A fatal yellow fever virus infection in China: description and lessons. Emerg Microbes Infect. 2016;5(7):e69. 10.1038/emi.2016.89 27406389 PMC5141266

[17] Ling Y Chen J Huang Q Hu Y Zhu A Ye S Yellow Fever in a Worker Returning to China from Angola, March 2016. Emerg Infect Dis. 2016;22(7):1317-8. 10.3201/eid2207.160469 27314417 PMC4918156

[18] Medlock JM Hansford KM Schaffner F Versteirt V Hendrickx G Zeller H A review of the invasive mosquitoes in Europe: ecology, public health risks, and control options. Vector Borne Zoonotic Dis. 2012;12(6):435-47. 10.1089/vbz.2011.0814 22448724 PMC3366101

[19] Miller BR Mitchell CJ Ballinger ME Replication, tissue tropisms and transmission of yellow fever virus in Aedes albopictus. Trans R Soc Trop Med Hyg. 1989;83(2):252-5. 10.1016/0035-9203(89)90667-6 2609379

[20] Miller BR Monath TP Tabachnick WJ Ezike VI Epidemic yellow fever caused by an incompetent mosquito vector. Trop Med Parasitol. 1989;40(4):396-9. 2623418

[21] Tesh RB Guzman H da Rosa AP Vasconcelos PF Dias LB Bunnell JE Experimental yellow fever virus infection in the Golden Hamster (Mesocricetus auratus). I. Virologic, biochemical, and immunologic studies. J Infect Dis. 2001;183(10):1431-6. 10.1086/320199 11319679

[22] Vainio J, Cutts F. *Yellow fever.* WHO/EPI/GEN/9811. Geneva: World Health Organization; 1998. p. 1–87.

[23] Fontenille D, Failloux AB, Romi R. Should we expect chikungunya and dengue in Southern Europe? In: Takken W, editor. Ecology and Control of Vector-Borne Diseases. Vol 1. Wageningen Academic Publishers; 2007. p. 169-84.

